# Acute and Chronic Whole-Body Vibration Exercise does not Induce Health-Promoting Effects on The Blood Profile

**DOI:** 10.1515/hukin-2015-0039

**Published:** 2015-07-10

**Authors:** Anastasios A. Theodorou, Vassilis Gerodimos, Konstantina Karatrantou, Vassilis Paschalis, Konstantina Chanou, Athanasios Z. Jamurtas, Michalis G. Nikolaidis

**Affiliations:** 1 Department of Physical Education and Sport Science, University of Thessaly, Trikala, Greece.; 2 Department of Health Sciences, European University Cyprus, Nicosia, Cyprus.; 3 Exercise Physiology and Biochemistry Laboratory, Department of Physical Education and Sport Sciences at Serres, Aristotle University of Thessaloniki, Serres, Greece.

**Keywords:** lipid profile, muscle damage, redox status, training, vibration, exercise

## Abstract

Whole-body vibration (WBV) exercise is an alternative, popular and easy exercise that can be followed by general public. Therefore, the aim of the present study was to investigate the influence of acute and chronic WBV exercise on health-related parameters. Twenty-eight women were allocated into a control group (n=11, mean ±SEM: age, 43.5 ±1.5 yr; body mass, 66.1 ±3.1 kg; height, 160.6 ±1.5 cm) and a vibration group (n=17, mean ±SEM: age, 44.0 ±1.0 yr; body mass, 67.1 ±2.2 kg; height, 162.5 ±1.5 cm). After baseline assessments, participants of the experimental group performed WBV training 3 times/week for 8 weeks. Before and after the chronic WBV exercise, the participants of the vibration group performed one session of acute WBV exercise. Blood chemistry measurements (hematology, creatine kinase, lactate dehydrogenase, aspartate aminotransferase, alanine aminotransferase, C-reactive protein, glucose, insulin, triacylglycerols, total cholesterol, high-density lipoprotein cholesterol, low-density lipoprotein cholesterol, apolipoprotein A1, apolipoprotein B and lipoprotein, thiobarbituric-acid reactive substances, protein carbonyls, total antioxidant capacity, uric acid, albumin and bilirubin) were assessed pre-exercise and post-exercise at the first and eighth week of WBV exercise in both control and vibration groups. The results failed to support any effect of both acute and chronic WBV exercise on biochemical health-related parameters. However, it seems that WBV exercise is a safe way of training without a negative impact on muscle and liver functionality.

## Introduction

Whole-body vibration (WBV) is a sophisticated type of exercise that has been arisen and became popular in sports training and fitness programs during the last decade (Cardinale and Wakeling, 2005). WBV exercise is performed using a vertically oscillating platform on which a person stands for a specific period of time. WBV exercise is categorized in two major modes depending on the transmission of vibration: i) side-alternating vibration stimulus and ii) synchronous vibration stimulus (Rittweger, 2010). In both vibration modes the variables determining the exercise intensity are the transmission, the frequency and the amplitude of vibration (Rittweger, 2010).

Whole body vibration embraces the major principles of the modern training regimes, based on which, training has to be efficient, short in duration, performed easier than traditional exercise modes (i.e. resistance and aerobic exercise) and, more importantly, has to cause favorable effects on human health and performance. WBV exercise is an alternative, popular and simple type of exercise that can be followed by the general public and the elderly (Bogaerts et al., 2009). Moreover, health centers advertise WBV exercise as the new revolutionary mode of exercise that may replace traditional resistance exercise. However, despite the steadily increasing popularity of WBV exercise, its effects on muscle performance are not clear. In fact, it has been found positive effects of acute or chronic WBV exercise on muscle performance (Bogaerts et al., 2009), whereas others have reported no effect on muscle performance (Gerodimos et al., 2010; Karatrantou et al., 2013), proprioception (Piecha et al., 2014) and on the excitability of the central and peripheral nervous system (Chmielewska et al., 2014).

Researchers also investigated the effects of WBV on health related parameters in both rats and humans. More specifically, chronic WBV training in rats was found to reduce body fat (Maddalozzo et al., 2008), enhance fracture healing (Leung et al., 2009), while decreasing bone resorption without affecting bone formation and bone mineral density (Nowak et al., 2014). Moreover, chronic WBV in rats caused positive alterations in immunological parameters and blood counts (Pawlak et al., 2013). Regarding humans, chronic WBV training was found to induce positive effects on knee-extensor maximal strength/explosiveness and flexibility in female athletes (Annino et al., 2007). Additionally, in healthy individuals chronic WBV exercise increased bone mineral density (Slatkovska et al., 2010), but had no effect on bone mineral density when applied in patients suffering from osteoporosis (Gomez-Cabello et al., 2014). Moreover, chronic WBV exercise in elderly enhanced neuromuscular performance (Furness and Maschette, 2009) and walking ability (Kawanabe et al., 2007). Additionally, an acute WBV was found to reduce plasma iterleukin-6 along with muscle soreness after downhill running (Broadbent et al., 2010), increased plasma free fatty acids (Goto and Takamatsu, 2005) and decreased plasma glucose concentration (Di Loreto et al., 2004).

It is clear that there is limited data regarding the effects of WBV on health related variables, thus there is a need for more comprehensive investigations in order to describe more precisely the effects of WBV exercise on various aspects of human biology. Considering the above, the aim of the present study was to investigate the influence of both acute and chronic WBV exercise on hematology, muscle and liver damage, systemic inflammation, insulin resistance, the lipid and apolipoprotein profile as well as on redox status.

## Material and Methods

### Participants

Twenty-eight healthy middle-aged women volunteered to participate in the present investigation. All subjects were asked to recall whether they had participated in regular resistance/aerobic training or in structured physical activity (including vibration exercise) for the last 3 months before the study entry. Individuals who reported participation in the aforementioned activities were excluded from the study. Volunteers were instructed to abstain from any strenuous exercise during their participation in the study as well as for five days prior to and following the two acute WBV exercise bouts. A written informed consent form to participate in the study was provided by all participants after they were informed about all risks, discomforts, and benefits involved in the study. Additionally, with regard to the subjects’ health condition a cardiologist provided a medical referral. The procedures were in accordance with the 1975 Declaration of Helsinki, as revised in 2000, and approval was received from the ethics committee of the University of Thessaly.

### Study design

Subjects were allocated into two groups: the control group (n=11, mean ±SEM: age, 43.5 ±1.5 yr; body mass, 66.1 ±3.1 kg; body height, 160.6 ±1.5 cm; BMI, 25.7 ±1.4) and the vibration group (n=17, mean ±SEM: age, 44.0 ±1.0 yr; body mass, 67.1 ±2.2 kg; body height, 162.5 ±1.5 cm; BMI, 25.5 ±0.9). During the first familiarization visit to the laboratory, participants stood on the vibration platform in a position similar to the one that they would use during the experiment. Two weeks later (week 1), participants of the vibration group performed an acute session of WBV exercise while participants of the control group assumed a similar position to that adopted by the vibration group without performing WBV. Subjects of both groups spend the same time on the platform. Blood samples were collected pre and post-exercise. Following the initial WBV session, the participants in the vibration group carried out eight weeks of vibration training consisting of three exercise sessions per week. Upon training completion, at week 8, volunteers who followed vibration training abstained from physical activities for 3 days. Afterwards, the participants of the vibration group performed an acute session of WBV exercise while the participants of the control group assumed a similar position to that adopted by the vibration group without performing WBV. Subjects of both groups spend the same time on the platform. Blood samples were also collected pre and post-exercise.

### Acute WBV exercise

In the present investigation, the medically certified Galileo Fitness WBV device was used (Novotec Medical Gmbh, Germany). The particular platform produces a side-to-side alternating vibration. The variables that determine the training load during WBV are the amplitude, frequency, and duration (Cardinale and Wakeling, 2005), while the interaction of the amplitude and frequency define the WBV acceleration (Bazett-Jones et al., 2008). The exercise protocol that was performed by the participants of the vibration group consisted of 6 min of WBV at frequency of 25 Hz with the 6 mm amplitude performed at the first and the last WBV exercise. The training load during WBV that was selected in the present investigation was based on findings of previous studies where a training load of 15–26 Hz frequency and the 3–6 mm amplitude was adequate for causing significant positive alterations in fitness and health related variables (Furness and Maschette, 2009; Gerodimos et al., 2010; Torvinen et al., 2002). Moreover, in the few studies that manipulated loading variables (frequency and/or amplitude) during side-to-side WBV exercise it was reported that higher frequencies and amplitudes could cause greater muscle activation compared to lower ones (Pollock et al., 2010; Ritzmann et al., 2010), which in turn may result in improvements in physical fitness and health. However, in the present investigation the highest frequency (30 Hz) and highest amplitude (8 mm) setting were avoided because our participants were untrained and had no previous experience in WBV training. In the present study, the maximum acceleration (amax) of vibration was 148 m/s^2^. For calculating the acceleration of the vibration the following equation was used: amax=a(2πf)2, where (f) is frequency and (a) is amplitude. The subjects of the control group assumed a standing position on the platform similar to that used by the subjects of the vibration group for the same time without performing WBV.

During the WBV exercise, participants were wearing sport shorts and non-slippery socks. The WBV amplitude was determined as the extent of the oscillatory motion (peak-to-peak displacement). More specifically, the participants’ right and left foot were placed on marks 3 of the platform that corresponded to the WBV amplitude of 6 mm. Their hands were touching the platform handles and the knees were maintained at the angle of 10° flexion (Gerodimos et al., 2010). The knee joint angle was monitored during each WBV exercise session using a goniometer (Gollehon, Lafayette). Exercise was performed between 7:00 and 10:00 a.m.. The subjects performed a warm-up for 7 min consisting of stationary cycling for 5 min (50 Watt, 50 rpm) and then stretching exercises for the torso and lower limbs (quadriceps, hamstring, tibialis anterior, calf, adductors, abductors). For the warm-up a common exercise (i.e., bicycling) of short duration and low intensity was chosen in order not to influence in any way the results of the intervention protocol.

### Chronic WBV exercise

The participants of the vibration group followed the training programme for 8 weeks (3 times/week). Three WBV sessions per week are considered an adequate training load that can induce positive effects on the fitness level and health related variables in untrained middle-aged individuals. The majority of studies that investigated the chronic effects of WBV training in middle-aged and elderly individuals implemented 2–3 training-sessions per week (Bautmans et al., 2005; Tapp and Signorile, 2014). The duration of the exercise (6–8 min) and frequency (20–25 Hz) of the vibration were gradually increased because the participants were untrained, middle-aged individuals and they had no previous experience in vibration exercise, so an abrupt exposure to high vibration frequency (25 Hz) could result in a disorientation of the participants. Regarding the gradually increased training load, in the first two weeks the duration of the exercise was 6 min (3 set × 2′ during the first week and 1 set × 6′ during the second week) and frequency was set at 20 Hz. In the next two weeks (3–4) the duration and frequency were 7 min (1 set × 7′) and 20 HZ, respectively. During week 5 and 6 the duration was 7 min (1 set × 7′) and frequency was 25 Hz. Finally during the last two weeks (7–8) the duration and frequency were 8 min (1 set × 8′) and 25 HZ, respectively. In every WBV session, the vibration frequency was gradually increased during the first minute until reaching the desirable frequency as previously suggested (Gerodimos et al., 2010). Amplitude was set at 6mm and participants’ position on the device was in accordance with that described during the acute WBV exercise. The exercise sessions were performed on Mondays, Wednesdays and Fridays or on Tuesdays, Thursdays and Saturdays. If a participant missed a session at the first scheduled day of the week (i.e., Monday or Tuesday) then she performed the session the next day and the following session was performed one day after the scheduled day. None of the participants missed or failed to accomplish a single training session.

### Blood collection and handling

Blood was collected in EDTA tubes, centrifuged immediately at 1,370 g for 10 min at 4 °C and the plasma was collected. Blood samples were stored in multiple aliquots at −80 °C and thawed only once before analysis. All blood samples were drawn in the morning after an overnight fast and all participants abstained from caffeine and alcohol for three days prior to sampling.

### Hematology

The hematological parameters were measured by a hematology analyzer (Beckman Coulter AcT-5, Nyon).

### Muscle and liver damage and inflammation

Creatine kinase (CK), lactate dehydrogenase (LDH), aspartate aminotransferase (AST), alanine aminotransferase (ALT) and C-reactive protein (CRP) were measured using an automatic chemistry analyzer (Hycel Lisa 200, France).

### Insulin resistance

Glucose was assayed using an enzymatic spectrophotometric method in an automatic chemistry analyzer (Hycel Lisa 200, Massy France). Plasma insulin was determined using an enzyme immunoassay kit from DRG (Marburg, Germany). The homeostasis model assessment (HOMA) was used as a surrogate measure of insulin resistance and was calculated as fasting insulin (μU×mL^−1^) × fasting glucose (mmol×L^−1^)/22.5.

### Lipid profile

Plasma triacylglycerols (TG) total cholesterol (TC), high-density lipoprotein cholesterol (HDLC), low-density lipoprotein cholesterol (LDLC), apolipoprotein A1, apolipoprotein B and lipoprotein (a) were measured using an automatic chemistry analyzer (Hycel Lisa 200, France). TC/HDLC (considered an atherogenic index) was also calculated.

### Redox status

Thiobarbituric-acid reactive substances (TBARS), protein carbonyls and total antioxidant capacity (TAC) were measured as previously described (Theodorou et al., 2010). Uric acid, albumin and bilirubin were measured using an automatic chemistry analyzer (Hycel Lisa 200, France). Each assay was performed in duplicates and within four months from the blood collection. A control sample was run in each assay. Each parameter was assayed in a single day to eliminate inter-assay variability.

### Statistical analysis

The distribution of all dependent variables was examined by the Shapiro-Wilk test and was found not to differ significantly from normality. Differences regarding physical characteristics between the groups at baseline were examined by an unpaired Student’s t-test. Three-way ANOVA [group (control or vibration) × training state (untrained or trained) × time (pre exercise and post exercise)] with repeated measurements were used to analyze the effect of WBV exercise on human health variables. If a significant interaction was obtained, pairwise comparisons were performed through the Sidak test. Post-exercise values of biochemical parameters measured were corrected for plasma volume changes. Data are presented as mean ± SEM. The level of significance was set at *α* = 0.05. The SPSS version 17.0 was used for all analyses (SPSS Inc., Chicago, Illinois).

## Results

### Physical characteristics

No differences were observed between the two groups regarding physical characteristics at baseline or at week 8.

### Hematology

The values of the hematological parameters measured are presented in Table 1. The levels of all hematological parameters were independent of the group, the training state or the time (Table 1).

### Muscle and liver damage and inflammation

Regarding CK, LDH, AST, ALT and CRP there was no significant main effect of the group, the training state and time or interactions among these factors (Table 2).

### Insulin resistance

**Figure 1:**
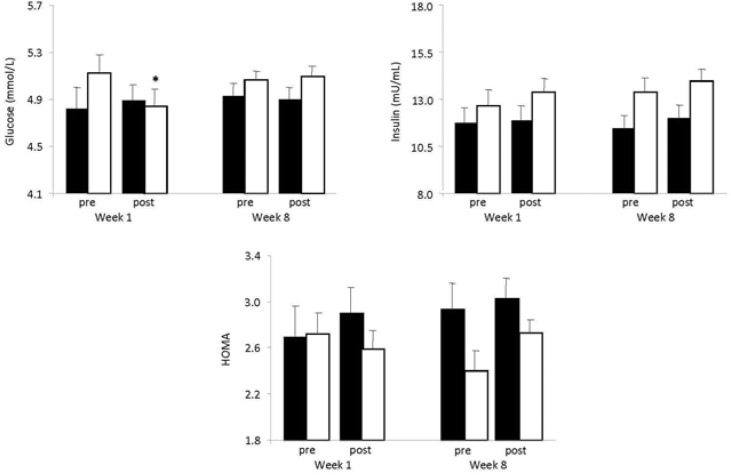
Blood insulin resistance indices (glucose, insulin, HOMA) at pre-exercise and post-exercise in the first and eighth week of WBV exercise in the control (closed bars) and the vibration group (open bars). ^*^Significantly different from the pre-exercise value in the same group (P < 0.05)

With regard to glucose, there was a significant group × training state × time interaction (p = 0.015; Figure 1). In the vibration group, glucose was significantly decreased post exercise after the first session at week 1 (p < 0.001; Figure 1). Concerning insulin and HOMA, there was no significant main effect of the group, the training state and time or interactions among these factors (Figure 1).

### Blood lipid profile

**Figure 2:**
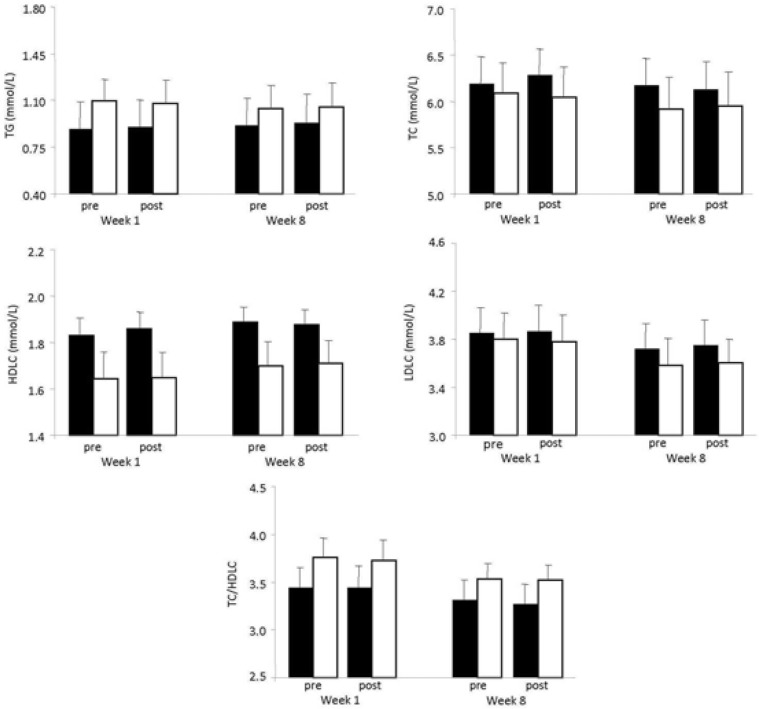
Blood lipid profile (TC, TG, HDLC, LDLC, TC/HDLC) at pre-exercise and post-exercise in the first and eighth week of WBV exercise in the control (closed bars) and the vibration group (open bars)

Regarding TG, TC, HDLC, LDLC and TC/HDLC there was no significant main effect of the group, the training state and time or interactions among these factors (Figure 2). Similarly, apolipoprotein-A1 and apolipoprotein-B did not change after acute or chronic exercise (Figure 2).

### Redox status

With respect to all the redox status indices, there was no significant main effect of the group, training state and time or interactions among these factors (Table 3).

## Discussion

To our knowledge, this is the first study that investigated the effect of chronic WBV exercise performed by adult healthy females on the blood lipid profile and oxidative stress. Based on the present data, acute and chronic WBV exercise failed to induce any effect on haematology, muscle and liver indices of damage, systemic inflammation, insulin resistance, blood lipid and apolipoprotein profile as well as on the blood redox status.

In the present investigation, the exercise frequency was set at 3 vibration bouts per week, which is a normal training routine, for 8 weeks. Previous studies that used WBV training of similar duration reported significant positive effects on fitness levels and health related variables (Annino et al., 2007; Furness and Maschette, 2009; Kawanabe et al., 2007; Slatkovska et al., 2010). Specifically, in two studies of the same nature it was reported that three WBV bouts per week for eight weeks were sufficient to induce improvements in knee-extensor maximal strength and flexibility in female athletes (Annino et al., 2007). Moreover, three WBV sessions per week for 6 weeks in elderly individuals resulted in improvements in neuromuscular performance (Furness and Maschette, 2009), while in elderly individuals, the walking ability was improved when they included in their training program one WBV session per week for two months (Kawanabe et al., 2007). Additionally, chronic WBV exercise may cause positive effects on bone mineral density in healthy individuals (Slatkovska et al., 2010), however, in patients suffering from osteoporosis, three WBV sessions per week for 11 weeks did not manage to induce any alterations in bone mineral density (Gomez-Cabello et al., 2014).

In the present investigation, the usage of WBV as an exercise stimulus was adopted because it is identical to the external forces (i.e., vibrations and oscillations) applied to tissues of the body during human movement/sporting activities (Cardinale and Wakeling, 2005). Moreover, the side-to-side vibration stimulus was used instead of the synchronous vibration stimulus because the vibration transmitted to the head during side-to-side vibration is significantly smaller (Abercromby et al., 2007). A commonly used WBV exercise protocol was adopted as it offers two major advantages: i) it can be easily followed and ii) no previous experience is needed. Generally, in the present investigation the amplitude, frequency, and duration of the exercise were chosen in order for untrained individuals, who were not previously familiarized with vibration exercise, to follow the intervention easily. Moreover, the configurations of the WBV exercise were previously used in studies of the same nature (Gerodimos et al., 2010; Karatrantou et al., 2013; Kawanabe et al., 2007; Rittweger et al., 2001). More specifically, 6 min duration of WBV exercise was used as it had been suggested that longer exposure to vibration exercise may elicit some negative effects like suppress the tonic vibration reflex and decrease muscle activation and force (Torvinen et al., 2002).

It is known that unaccustomed exercises of excessive volumes or intensities may provoke muscle damage and muscle soreness (Paschalis et al., 2007). Lately, increasing evidence suggests that applying a short duration WBV protocol before and/or after muscle-damaging exercise is beneficial for attenuating muscle soreness and inflammation (Broadbent et al., 2010). On the other hand, there are some concerns in the literature that WBV exercise can provoke muscle injury and consequently induce elevation of muscle damage indices (Gojanovic et al., 2011). The results of the present investigation failed to support any effect of acute or chronic WBV exercise on muscle and liver damage as well as on systemic inflammation. However, it is worth noticing that indices of muscle damage may peak at 48 h post exercise (Paschalis et al., 2007), while in the present investigation measurements on the days following bouts of WBV exercise were not performed.

It is widely known that there is increased glucose uptake during dynamic exercise which can be attributed to the acute effect of exercise on glucose metabolism and/or to the chronic adaptations induced by exercise (Rose and Richter, 2005). In the present study, a decrease in the glucose level after the first exercise session in the WBV group was found, which is in line with the findings of a previous study of the same nature (Di Loreto et al., 2004). Indeed, Di Loreto et al. (2004) found that a vibration session caused decline in plasma glucose concentration. The decreased levels of glucose after WBV could be attributed to the increased muscle activity during WBV exercise (Hazell et al., 2010). On the other hand, the absence of glucose alteration after WBV at the 8th week could be attributed to the muscle adaptation to this type of exercise. Insulin and HOMA were not altered at any time point of assessments.

Positive alterations in the blood lipid profile are among the major health benefits of regular endurance and resistance exercise (Booth et al., 2000). However, in the present investigation, both acute and chronic WBV exercise did not manage to cause any changes in TG, TC, HDLC, LDLC, apolipoprotein A1 or apolipoprotein B. Taking into account i) the mild physiological stress imposed by WBV exercise, ii) the limited effort required by the subjects during WBV exercise, iii) the relatively short total duration of WBV exercise and iv) the restrictions of WBV frequency steered by manufacturers’ recommendations, the absence of any effect on the lipid profile was an expected outcome. Indeed, in a number of investigations it was concluded that WBV alone, probably cannot favourably affect aerobic fitness, since elevations in energy turnover and cardiorespiratory responses are only mild to moderate (Rittweger et al., 2001). Moreover, it is known that under various conditions (i.e., standing or squatting, with or without an additional load) the vibration-specific energy turnover at 26 Hz frequency amounts to about 4.5 ml×min-1×kg-1 (Rittweger et al., 2001), whereas for comparison, the oxygen uptake at rest is approximately 3.5 ml×min-1×kg-1.

Oxidative stress is defined as an increase in the level of reactive species and/or oxidant biomarkers (Nikolaidis et al., 2012). Oxidative stress constitutes a ubiquitous fundamental biological response to the alteration of redox homeostasis imposed by exercise (Nikolaidis et al., 2012). Exercise-induced oxidative stress appears after any type of exercise provided that the intensity and/or duration are of a sufficient level (Fisher-Wellman and Bloomer, 2009). The fact that the WBV protocol employed failed to induce any alteration in the redox biomarkers measured corroborates the absence of effect on muscle damage, inflammation, insulin sensitivity and the lipid profile.

Based on the findings of the present investigation, it seems that WBV is a safe way of exercise without negative impact on muscle and liver functionality. On the other hand, with WBV exercise it is unlikely for someone to achieve the beneficial changes that accompany regular aerobic and/or resistance exercise (e.g., increased insulin sensitivity and/or favourable alterations in the lipid profile). This is probably because WBV exercise only mildly increases the energy expenditure causing a limited physical stress.

On the other side, elderly and physically disabled people that have difficulties participating in aerobic and/or resistance training activities could potentially benefit from WBV exercise (Chanou et al., 2012). Indeed, evidence has shown that WBV training in physically impaired individuals (such as elderly or people suffering from a chronic disease) improves muscle strength (von Stengel et al., 2012), postural control (Bogaerts et al., 2007), balance and stability (Bautmans et al., 2005) and decreases fall risk (Bruyere et al., 2005). Moreover, WBV training was found to induce positive effects on bone mineral density in healthy individuals (Slatkovska et al., 2010) but did not have any effect when applied in patients suffering from osteoporosis (Gomez-Cabello et al., 2014). Additionally, acute WBV was found to reduce plasma iterleukin-6 (inflammation factor) and muscle soreness after downhill running (Broadbent et al., 2010). WBV exercise increased the level of plasma free fatty acids (Goto and Takamatsu, 2005), as a result of the increase of growth hormone which is known to have a powerful lipolytic effect (Gravholt et al., 1999).

In conclusion, it is clear that regular WBV exercise for eight weeks cannot induce health-promoting effects in healthy individuals. Traditional forms of physical activities, such as aerobic or resistance exercise, remain the most important physiological tool in order to achieve and maintain a healthy status.

## Figures and Tables

**Table 1:** Hematologic variables in the control and vibration group pre and post exercise (mean ±SEM)

	Control (week 1)		Vibration (week 1)		Control (week 8)		Vibration (week 8)	
			
	pre	post	pre	post	pre	post	pre	post
Hct (%)	38.2 ± 0.8	38.2 ± 0.8	38.6 ± 0.7	39.2 ± 0.7	38.0 ± 0.8	38.2 ± 0.8	38.0 ± 0.8	38.4 ± 0.7
Hb (g·dL^−1^)	12.5 ± 0.3	12.5 ± 0.3	12.5 ± 0.3	12.9 ± 0.3	12.3 ± 0.2	12.6 ± 0.3	12.4 ± 0.3	12.6 ± 0.3
RBC (10^12^·L^−1^)	4.6 ± 0.1	4.6 ± 0.1	4.8 ± 0.1	4.9 ± 0.1	4.6 ± 0.1	4.6 ± 0.1	4.8 ± 0.1	4.9 ± 0.1
MCV (fL)	84.1 ± 2.1	84.1 ± 2.1	80.9 ± 2.8	80.9 ± 2.8	83.5 ± 1.9	83.3 ± 1.9	80.6 ± 2.9	80.4 ± 2.9
MCH (pg·cell^−1^)	27.5 ± 0.8	27.4 ± 0.8	26.3 ± 1.1	26.4 ± 1.1	27.0 ± 0.8	27.0 ± 0.7	26.6 ± 1.1	26.4 ± 1.1
MCHC (g·dL^−1^)	32.7 ± 0.1	32.6 ± 0.2	32.4 ± 0.2	32.5 ± 0.3	32.7 ± 0.1	32.6 ± 0.2	32.8 ± 0.3	32.7 ± 0.3
RDW(%)	14.2 ± 0.6	14.0 ± 0.5	14.5 ± 0.5	14.5 ± 0.5	14.2 ± 0.5	13.9 ± 0.5	14.6 ± 0.6	14.6 ± 0.5
WBC (10^9^·L^−1^)	6.5 ± 0.7	6.5 ± 0.7	7.1 ± 0.4	7.5 ± 0.4	6.4 ± 0.5	6.5 ± 0.5	7.6 ± 0.4	7.7 ± 0.4
Neu (%)	45.8 ± 2.6	47.0 ± 2.8	54.1 ± 1.8	55.2 ± 1.8	52.5 ± 1.5	51.9 ± 1.4	51.8 ± 1.7	52.2 ± 1.6
Lymph (%)	38.4 ± 1.5	39.2 ± 1.7	35.0 ± 1.9	34.1 ± 1.8	36.2 ± 1.6	37.6 ± 1.5	37.2 ± 1.7	36.8 ± 1.7
Mono (%)	8.2 ± 0.4	7.9 ± 0.3	7.9 ± 0.4	7.6 ± 0.3	8.5 ± 0.4	8.0 ± 0.4	8.1 ± 0.5	7.6 ± 0.5
EO (%)	2.0 ± 0.2	2.1 ± 0.2	2.5 ± 0.3	2.5 ± 0.3	2.3 ± 0.3	2.0 ± 0.2	2.4 ± 0.3	2.3 ± 0.3
Baso (%)	0.6 ± 0.1	0.5 ± 0.1	0.6 ± 0.1	0.6 ± 0.1	0.5 ± 0.1	0.5 ± 0.1	0.6 ± 0.1	0.6 ± 0.1
Plt (10^9^·L^−1^)	260 ± 10	259 ± 10	297 ± 18	213 ± 19	267 ± 12	268 ± 14	283 ± 18	292 ± 18
MPV (fL)	9.7 ± 0.2	9.6 ± 0.2	8.8 ± 0.2	9.1 ± 0.2	9.5 ± 0.3	9.6 ± 0.3	9.0 ± 0.2	9.0 ± 0.2

EO, Eosinophils; Hb, hemoglobin; Hct, hematocrit; Lymph, lymphocytes; MCH, mean cell hemoglobin; MCHC, mean cell hemoglobin concentration; MCV, mean cell volume; Mono, Monocytes; MPV, Mean platelet volume; Neu, neutrophils; Plt, platelets; RBC, red blood cells; RDW, red blood cell distribution width; WBC, white blood cells.

**Table 2:** Muscle and liver and inflammation variables in the control and vibration group pre and post exercise (mean ±SEM)

	Control (week 1)		Vibration (week 1)		Control (week 8)		Vibration (week 8)	
			
	pre	post	pre	post	pre	post	pre	post
CK (U/L)	153 ± 22	159 ± 21	153± 29	141 ± 27	123 ± 9	123 ± 12	103 ± 11	110 ± 13
LDH (U/L)	164± 10	163 ± 11	157 ± 11	152 ± 11	159 ± 10	154 ± 8	143 ± 8	145 ± 8
AST (U/L)	23.3 ± 2.6	23.3 ± 2.3	21.8 ± 1.7	21.3 ± 1.9	24.3 ± 2.6	24.1 ± 2.4	23.7 ± 1.2	23.1 ± 1.1
ALT (U/L)	25.4 ± 2.2	25.1 ± 2.1	24.3 ± 1.6	23.8 ± 1.3	24.0 ± 1.9	23.8 ± 1.6	25.2 ± 1.3	24.4 ± 1.6
CRP (mg/L)	0.22 ± 0.10	0.24 ± 0.11	0.16 ± 0.06	0.17 ± 0.05	0.20 ± 0.06	0.18 ± 0.06	0.11 ± 0.02	0.12 ± 0.02

ALT, alanine aminotransferase; AST, aspartate aminotransferase; CK, creatine kinase; CRP, C-reactive protein; LDH, lactate dehydrogenase.

**Table 3:** Redox status in the control and vibration group pre and post exercise (mean ±SEM)

	Control (week 1)		Vibration (week 1)		Control (week 8)		Vibration (week 8)	
			
	pre	post	pre	post	pre	post	pre	post
TBARS (μM)	10.2 ± 0.7	10.8 ± 0.8	11.3± 0.7	11.5 ± 0.6	10.7 ± 0.6	10.9 ± 0.5	11.9 ± 0.5	12.4 ± 0.6
Carbonyls (nmol/mg pr.)	0.40 ± 0.04	0.42 ± 0.04	0.43 ± 0.03	0.39 ± 0.03	0.48 ± 0.04	0.46 ± 0.04	0.49 ± 0.04	0.42 ± 0.02
TAC (mM DPPH)	0.42 ± 0.03	0.39 ± 0.04	0.47 ± 0.04	0.44 ± 0.02	0.40 ± 0.05	0.48 ± 0.04	0.48 ± 0.03	0.53 ± 0.04
Albumin (g/L)	44.9 ± 0.6	45.5 ± 0.6	46.2± 0.5	46.0 ± 0.6	45.4 ± 0.7	45.0 ± 1.1	44.2 ± 0.5	44.8 ± 0.6
Billirubin (μM)	9.8 ± 0.5	9.9 ± 0.8	12.5± 1.5	11.9 ± 1.4	10.3 ± 0.8	9.8 ± 0.9	10.9 ± 1.2	11.4 ± 1.2
Uric Acid (μM)	0.38 ± 0.05	0.34 ± 0.05	0.34 ± 0.03	0.29 ± 0.04	0.38 ± 0.04	0.37 ± 0.04	0.35 ± 0.03	0.36 ± 0.04

TBARS, thiobarbituric-acid reactive substances; TAC, total antioxidant capacity
